# The impact of the COVID-19 pandemic on health services utilization in China: Time-series analyses for 2016–2020

**DOI:** 10.1016/j.lanwpc.2021.100122

**Published:** 2021-03-24

**Authors:** Hong Xiao, Xiaochen Dai, Bradley H. Wagenaar, Fang Liu, Orvalho Augusto, Yan Guo, Joseph M Unger

**Affiliations:** a Public Health Sciences Division, Fred Hutchison Cancer Research Center, Seattle, WA, United States; b School of Public Health, Zhejiang University, Hangzhou, China; c Department of Health Metrics Sciences, University of Washington, Seattle, WA, United States; d Institute for Health Metrics and Evaluation, University of Washington, Seattle, WA, United States; e Department of Global Health, University of Washington, Seattle, WA, United States; f Department of Epidemiology, University of Washington, Seattle, WA, United States; g Chinese Center for Disease Control and Prevention, Beijing, China; h Universidade Eduardo Mondlane, Maputo, Mozambique; i Department of Global Health, School of Public Health, Peking University, Beijing 100191, China

## Abstract

**Background:**

The aim of this study is to quantify the effects of the SARS-CoV-2 pandemic on health services utilization in China using over four years of routine health information system data.

**Methods:**

We conducted a retrospective observational cohort study of health services utilization from health facilities at all levels in all provinces of mainland China. We analyzed monthly all-cause health facility visits and inpatient volume in health facilities before and during the SARS-CoV-2 outbreak using nationwide routine health information system data from January 2016 to June 2020. We used interrupted time series analyses and segmented negative binomial regression to examine changes in healthcare utilization attributable to the pandemic. Stratified analyses by facility type and by provincial Human Development Index (HDI) – an area-level measure of socioeconomic status – were conducted to assess potential heterogeneity in effects.

**Findings:**

In the months before the SARS-CoV-2 outbreak, a positive secular trend in patterns of healthcare utilization was observed. After the SARS-CoV-2 outbreak, we noted statistically significant decreases in all indicators, with all indicators achieving their nadir in February 2020. The magnitude of decline in February ranged from 63% (95% CI 61–65%; *p*<0•0001) in all-cause visits at hospitals in regions with high HDI and 71% (95% CI 70–72%; *p*<0•0001) in all-cause visits at primary care clinics to 33% (95% CI 24–42%; *p*<0•0001) in inpatient volume and 10% (95% CI 3–17%; *p* = 0•0076) in all-cause visits at township health centers (THC) in regions with low HDI. The reduction in health facility visits was greater than that in the number of outpatients discharged (51% versus 48%; *p*<0•0079). The reductions in both health facility visits and inpatient volume were greater in hospitals than in primary health care facilities (*p*<0•0001) and greater in developed regions than in underdeveloped regions (*p*<0•0001). Following the nadir of health services utilization in February 2020, all indicators showed statistically significant increases. However, even by June 2020, nearly all indicators except outpatient and inpatient volume in regions with low HDI and inpatient volume in private hospitals had not achieved their pre-SARS-COV-2 forecasted levels. In total, we estimated cumulative losses of 1020.5 (95% CI 951.2- 1089.4; *P*<0.0001) million or 23.9% (95% CI 22.5–25.2%; *P*<0.0001) health facility visits, and 28.9 (95% CI 26.1–31.6; *P*<0.0001) million or 21.6% (95% CI 19.7–23.4%; *P*<0.0001) inpatients as of June 2020.

**Interpretation:**

Inpatient and outpatient health services utilization in China declined significantly after the SARS-CoV-2 outbreak, likely due to changes in patient and provider behaviors, suspension of health facilities or their non-emergency services, massive mobility restrictions, and the potential reduction in the risk of non-SARS-COV-2 diseases. All indicators rebounded beginning in March but most had not recovered to their pre-SARS-COV-2 levels as of June 2020.

**Funding:**

The National Natural Science Foundation of China (No. 72042014).

**Research in Context**

**Evidence before this study**

The SARS-CoV-2 pandemic has had profound effect on the performance of health care systems throughout affected countries. We searched PubMed and Google Scholar with the search terms “COVID-19″, “SARS-COV-2″, “impact”, “effect”, “health”, “services”, “utilization”, “admission”, “hospitalization” in both English and Chinese up to Nov 1, 2020. We found one peer-reviewed published report using nationally representative registry data about the effect of the COVID-19 pandemic on stroke care in China, showing a 25% reduction in thrombolysis and thrombectomy cases in February 2020 as compared with February 2019. Data from the USA, France and Italy documented decreases in imaging utilizations, hospital admissions and hospitalizations for cardiovascular and cerebrovascular diseases. Most studies relied on small samples and used data collected at single timepoints before and during the outbreak, and were unable to systematically describe long-term trends. Additionally, these studies did not present comprehensive data on heterogeneity of effects across subnational regions and types of health facilities.

**Added value of this study**

Our study is the first systematic analysis of all-cause health facility visits and inpatient volumes before and during the pandemic in China. We extended existing analyses to cover a census of all types of health facilities in China over a 54-month period from January 2016 to June 2020. We estimated that national health facility visits and inpatients volume decreased by 23.9% and 21.6% respectively from January to June 2020. We found more marked reductions in hospitals than in primary health care settings; moreover, reductions were greater in developed regions than in underdeveloped regions. Inpatient and outpatient healthcare services have not recovered to their pre-pandemic levels as of June 2020. These findings add to the body of literature demonstrating that the COVID-19 pandemic had significant negative effects on health services utilization. The results also provide insights about how health services have evolved since the SARS-COV-2 outbreak in China.

**Implications of all the available evidence**

Both our findings and those of previous studies show how the COVID-19 pandemic has had a devastating collateral effect on health services utilization, irrespective of regional variation in the severity of the pandemic. Although utilizations of health services are improving since control of the pandemic was achieved, utilization rates have not yet returned to their pre-pandemic levels. These findings call for awareness of the risk of patients deferring or forgoing health services, and targeted interventions to provide appropriate public health information to prevent and mitigate the negative effects in the case of a continued pandemic or similar epidemics in the future. The potential increased use of telehealth and virtual care as substitutes for in-person visits during the COVID-19 pandemic should be monitored and evaluated to help guide regulatory actions and to aid the tracking of healthcare utilization.

Alt-text: Unlabelled box

## 2 Introduction

Responding to an outbreak of SARS-COV-2 in December 2019, China implemented a range of behavioral and clinical interventions to mitigate the epidemic in China and worldwide. These unprecedented interventions included large-scale diagnostic testing, rapid isolation of suspected and confirmed cases and their contacts, comprehensive contact tracing, and large-scale stringent restrictions on mobility.

In addition to banning travel to and from Wuhan city on January 23rd 2020, all provinces, municipalities and autonomous regions in mainland China subsequently activated the Level-I response to public health emergencies, the highest level of such responses, within one week [1], [2], [3], [4], [5]. As part of the emergency response, consistent guidance on managing the pandemic was implemented, intracity public transport suspended, intercity travel prohibited, public gathering banned, schools and entertainment venues closed, and information widely disseminated in a timely manner [2,^3^,[6], [7], [8].

Healthcare utilization has repeatedly been observed to decrease during pandemics, due to such factors as mobility restrictions, social distancing measures, and fears of contracting the virus within health facilities, as patients and healthcare providers defer or forego routine healthcare, especially elective and preventive visits [9], [10], [11], [12], [13], [14], [15], [16], [17], [18], [19], [20]. The magnitude of the impact of the pandemic have varied markedly according to the type of healthcare service, location, and type of facility [11,^12^,[14], [15], [16],[21], [22], [23], [24], [25], effects that have exacerbated existing inequities in the health system. In a recent example, analyses using routine health information system data from Guinea, Liberia and Sierra Leone showed substantial reductions in the delivery of maternal, child and reproductive health services, disruptions in HIV and tuberculosis testing, and large-scale collapse of vaccine and malaria case management programs during the 2014–2015 Ebola virus disease outbreak [9,^17^,^26^,27]. Most primary health care indicators in Liberia recovered to pre-Ebola levels one year after the end of the outbreak, although a similar rapid recovery was not observed in Guinea [9,17]. Significant reductions in admissions, diagnoses and treatments of stroke and acute myocardial infarction have been reported in China, France, Italy and America early in the COVID-19 pandemic [12,^14^,^15^,^22^,28]. Notable decreases in enrollment in non- SARS-COV-2 clinical trials were found despite proactive steps taken to allow for greater flexibility such as remote consent and virtual visits [29]. To our knowledge, no study has comprehensively examined the impact of the COVID-19 pandemic on national healthcare services utilization and the resumption of services after the pandemic in China.

The aim of the present study is to examine the impact of the SARS-COV-2 outbreak on outpatient and inpatient services utilization across China. We also investigated whether trends in utilization differed by area-level socioeconomic status (SES), category of health facility and treatment setting.

## 3 Methods

### 3.1 Data sources and outcomes

We extracted all available data for monthly facility-based health services utilization from the routine health information system of the Center for Health Statistics and Information, National Health Commission of China for the period from January 2016, to June 2020. Since 2007, all types of health facilities at all levels except for village clinics and primary care clinics have been required to directly report monthly health services utilization to the Health Commissions of the 31 provinces, municipality and autonomous regions in mainland China (except Hong Kong and Macau) via the provincial level electronic direct-reporting platforms. County-level Health Bureaus are responsible for collecting utilization data from village clinics and primary care clinics and then reporting the data directly to Provincial Health Commissions on a monthly basis. Provincial Health Commissions verify and review the data at the primary level, investigate missing report, aggregate the data and report to the National Health Commission. The National Health Commission oversees and checks the quality of data at the secondary level, and also provides technical training and support to staff at the provincial and county level involved in the reporting process [30]). The primary indicators were the monthly number of health facility visits (outpatient and emergency department visits) and number of inpatients discharged by region and/or by health facility type nationwide. Monthly utilization counts for hospital or township health center (THC), a subtype of primary health care facility (PHCF), were available at the provincial level. Data for hospitals (public/private; first/secondary/tertiary/undefined level), other subtypes of PHCF (community health center, primary care clinic and village clinic) and other health facilities were aggregated at the national level. Subnational level human development index (HDI), a summary area-level measure of life expectancy, education and income level, was retrieved from the China National Human Development Report 2019 to reflect regional-level SES. A key advantage of examining an area-level measure in this context is its utility in providing evidence to help guide community-level interventions and policies. Each province, municipality and autonomous region was categorized into one of the three (high, middle and low) SES groups based on its HDI. Yearly urban and rural population estimates by region were extracted from China Statistical Yearbooks. This study was exempt from institutional review oversight since the data are publicly available and aggregated at population level.

Our aim was to examine a series of three interrelated research questions, including: (1) Was the SARS-CoV-2 outbreak associated with changes in outpatient and inpatient services utilization? We hypothesized that the outbreak resulted in meaningful reductions in utilization; (2) Did potential changes in healthcare services utilization due to the SARS-CoV-2 outbreak differ by regional-level SES, health facility type and treatment setting within health facility? We hypothesized that heterogeneity in the magnitude of relative changes in utilization existed across regions with different SES, various types of health facilities at different levels, and between outpatient and inpatient care setting; (3) Did changes in healthcare services utilization associated with the SARS-CoV-2 outbreak return to pre-pandemic patterns after the pandemic, and did patterns of recovery differ by regional SES, facility type and treatment setting? We hypothesized that healthcare utilization rates recovered after the peak of the pandemic in China- though to lower rates than before the pandemic – and that but the extent of recovery differed by regional SES, facility type and treatment setting.

### 3.2 Statistical analyses

We conducted an interrupted time series analysis to examine the impact of SARS-COV-2 on healthcare services utilization [31,32]. The impact was modeled using a segmented negative binomial regression with pre-SARS-COV-2 trends (January 2016 - December 2019) and indicator variables for each month in post-SARS-COV-2 period (January-June 2020). Due to known heterogeneity in the levels as well as the long-term trends of healthcare utilization across provinces, we employed a mixed-effects model with random intercepts and random slopes over time for province in subnational analyses. To adjust for observed seasonal patterns and the effect of the spring festival, we included fixed-effect monthly indicator variables and number of spring festival days as covariates. Comparison of changes in healthcare utilization between regions with different SES were conducted using interaction tests [33], [34], [35]. Prior to the conduct of regression analyses, we analyzed data over-dispersion using likelihood ratio tests and confirmed that negative binomial models were more appropriate than Poisson equivalents, as the negative binomial model represents Poisson regression adjusted for excess variation [36]. The negative binomial model equation estimating monthly utilization at provincial level was expressed as follows:

$$\begin{array}{ccc} \text{ln}\;\left(E\left(Y_{\mathrm{it}}\right)\right) & = & \beta_{0i}\;+\beta_{1i}\;T_{t}\;+\sum_{j=1}^{6}\beta_{2j}\;T_{j}+\beta_{3}\;\mathrm{HD}I_{i}\\ & & +\;\sum_{j=1}^{6}\beta_{4j}\;\mathrm{HD}I_{i}*T_{j}\;+\beta_{5}SF_{\;t}+\beta_{6}\;\left(SF_{t}*\mathrm{HDI}\right)\;\\ & & +\;\sum_{m=2}^{12}\beta_{\mathrm{Mm}}\;\mathrm{Month}+\mathrm{offs}\mathrm{et}\left(\ln\left(P_{\mathrm{it}}\right)\right) \end{array}$$

Here, *Y_it_* denotes the value of one of the response variables (e.g., monthly outpatient visits at hospitals) included in this study for province *i* at time *t. T_t_* is the time (months) elapsed since the start of the study. *T_j_* is the indicator of the post SARS-COV-2 month *j* (from January to June 2020)*. HDI_i_* is the SES category (low (reference group), middle or high) that province *i* is allocated to. *SF_t_* is the number of days of spring festival holiday in month t. *Month* is the indicator of calendar month of the year with month of January as the reference category. *P_it_* is the catchment population for hospitals or THCs in province *i* at time *t*. β_0_*_i_* represents the model intercept with both a fixed effect and province-level random effects, and β_1_*_i_* represents the underlying pre-SARS-COV-2 secular trends with both a fixed effect and province-level random effects. The incidence rate ratio (IRR, model-fitted versus model-expected/counterfactual level had the pandemic not occurred) of utilization in month j of the post-SARS-COV-2 period for the low HDI group was quantified as exp(β_2_*_j_*). The ratio of IRR, quantified as exp(β_4_*_j_*), compares the IRR of a specific HDI group with that of the low HDI group in month j of the post-SARS-COV-2 period. Tibet and Hubei were excluded from the subnational analysis due to missing data in the pre- and post-outbreak (January and February 2020) period respectively. No missing data points or extreme outliers were found in other provinces after visually inspecting the data by indicator, health facility type and region. Each type of health facility (hospital or THC) and healthcare utilization (inpatient visits or inpatients discharged) was analyzed separately.

We employed fixed-effects models to analyze the impact of the pandemic on healthcare utilization at each subtype of healthcare facility. Comparison between different subtypes of facility were conducted using interaction tests. Potential effects of seasonality, the spring festival and catchment population were also examined. An AR [1] correlation structure and Newey-West Heteroskedasticity and Autocorrelation Consistent (HAC) standard errors were used in mixed-effects and fixed-effect models respectively to accommodate serial autocorrelation in residual errors, per results of the PACF and Cumby-Huizinga general test [37]. Lost health services utilizations were calculated as the differences between the mean fitted values under the full model and the forecasted (counterfactual) model assuming that the pandemic did not occur. To compute 95% confidence intervals around these differences, we used the range from the percentiles 2.5 and 97.5 of 10,000 sets of predictions per month under each model using the coefficients and multivariate normal distributions of each model [17]. P-values were calculated as the smaller of the proportion of simulated values falling either above or below zero. This value was then multiplied by 2 to represent a 2-sided p-value [17]. We followed STROBE reporting guideline for retrospective observational cohort study [38]. Analyses were conducted in R-version 4.0.2. Statistical significance was set at alpha=0.05 and all tests were two-sided.

## 4 Role of the funding source

The funder of the study had no role in study design, data collection, data analysis, data interpretation, or writing of the report. The corresponding author had full access to all the data in the study and had final responsibility for the decision to submit for publication.

## 5 Results

**Fig. 1:**
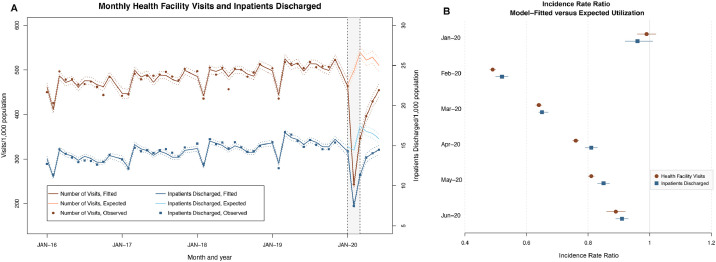
**Monthly health care services utilization rate over time.** Panel A) Monthly health facility visits and inpatients discharged per 1000 population over time. The dark red dots show observed rates, the solid dark red line indicates the model-fitted rates and the solid light red line represents the model-based expected (or counterfactual level had the pandemic not occurred) rates for health facility visits. Similarly, the dark blue squares show observed rates, the solid dark blue line indicates the model-fitted rates and the solid light blue line represents the model-based expected (or counterfactual) rates for inpatients discharged. The dashed lines are 95% confidence intervals around the fitted mean. The gray rectangular shaded area represents the period when over 95% of SARS-COV-2 cases were diagnosed during the study period. Panel B) The ratio of fitted versus expected utilization rate. The red dots and blue square show estimates for health facility visits and inpatients discharged, respectively.

The total number of health facilities in the analysis increased from 950, 216 (27, 795 hospitals, 922, 421 PHCFs) in January 2016 to 997, 234 (34, 658 hospitals, 962, 576 PHCFs) in June 2020, serving a total population of approximately 1.4 billion (urban 830 million, rural 560 million). Data showed strong and consistent increases in monthly health facility visits and inpatients discharged across the pre-COVID period. The monthly all cause visits and inpatients discharged increased by 18% (110, 505, 000/618, 623, 000) and 26% (4, 436, 000/21, 454, 000) respectively from January 2016 to December 2019. All indicators exhibited significant seasonal patterns, with the highest and lowest volume in utilizations occurring in March and February respectively (Table 1; Fig. 1; Fig. S1). 83, 472 cases of SARS-COV-2 occurred nationwide (including 1 case in Tibet and 68, 005 in Hubei) from 31 Dec 2020 to 30 June 2020. The number of confirmed cases outside Tibet and Hubei ranged from 18 (0.1%) in Qinghai to 1, 641 (10.6%) in Guangdong province. Fourteen percent, 82% and 4% of confirmed cases were reported in January, February and from March to June 2020 respectively.

**Table 1:** Monthly number of health facility visits and inpatients discharged from January 2016 through June 2020, stratified by health facility type.

	Year 2016–20,19^2^												Year 2020											
	Jan		Feb		Mar		April		May		June		Jan Initial Pandemic period		Feb Pandemic period		Mar Initial Reopening Period		April Initial Reopening Period		May Initial Reopening Period		June Initial Reopening Period	
	Mean Obs. (10, 000)	Growth rate^1^ (%)	Mean Obs. (10, 000)	Growth rate^1^ (%)	Mean Obs. (10, 000)	Growth rate^1^ (%)	Mean Obs. (10, 000)	Growth rate^1^ (%)	Mean Obs. (10, 000)	Growth rate^1^ (%)	Mean Obs. (10, 000)	Growth rate^1^ (%)	Obs. (10, 000)	Growth rate^1^ (%)	Obs. (10, 000)	Growth rate^1^ (%)	Obs. (10, 000)	Growth rate^1^ (%)	Obs. (10, 000)	Growth rate^1^ (%)	Obs. (10, 000)	Growth rate^1^ (%)	Obs. (10, 000)	Growth rate^1^ (%)
*Number of visits*																								
All health facilities	65,598	−2.7	60,368	−8.0	69,673	15.4	67,962	−2.5	68,786	1.2	66,366	−3.5	64,702	−11.3	33,894	−47.6	48,311	42.5	55,292	14.4	59,929	8.4	63,470	5.9
Hospital	27,143	−3.1	23,986	−11.6	30,119	25.6	28,993	−3.7	29,730	2.5	28,881	−2.9	26,802	−15.9	12,492	−53.4	21,458	71.8	25,126	17.1	27,187	8.2	28,720	5.6
Primary Health Care facility	36,125	−2.3	34,294	−5.1	36,060	5.1	36,413	1.0	36,410	0.0	36,136	−0.8	35,669	−6.8	20,302	−43.1	25,003	23.2	28,098	12.4	30,423	8.3	32,278	6.1
Other Health facility	2230	−12.8	2087	−6.4	2594	24.3	2556	−1.5	2774	8.5	2574	−7.2	2231	−19.0	1100	−50.7	1849	68.1	2068	11.8	2320	12.2	2471	6.5
*Inpatients Discharged*																								
All Health Facility	1964	1.6	1689	−14	2140	26.7	2075	−3.0	2042	−1.6	1960	−4.0	1996	−7.0	1043	−47.7	1584	51.9	1886	19.1	1962	4.0	2020	3.0
Hospital	1545	2.5	1302	−15.7	1668	28.1	1612	−3.4	1600	−0.7	1537	−3.9	1624	−5.9	783	−51.8	1239	58.2	1512	22.0	1579	4.4	1636	3.6
Primary Health Care facility	332	−2.1	302	−9.0	386	27.8	376	−2.6	355	−5.5	337	−5.2	285	−12.4	198	−30.5	274	38.4	306	11.7	313	2.3	311	−0.5
Other Health facility	86	−5.5	76	−11.6	87	14.5	86	−1.1	89	3.1	86	−3.6	86	−8.2	63	−26.8	71	12.7	69	−3.0	71	2.3	73	4.0

1 Growth rate: unadjusted growth rate compared with previous month.

2 Monthly observed estimates for the years 2016–2019 represent the mean number for a given month over the 4 year period. Visits include outpatient and emergency department visits.

### 5.1 Unadjusted changes in utilization rate during the COVID-19 pandemic

**Fig. 2:**
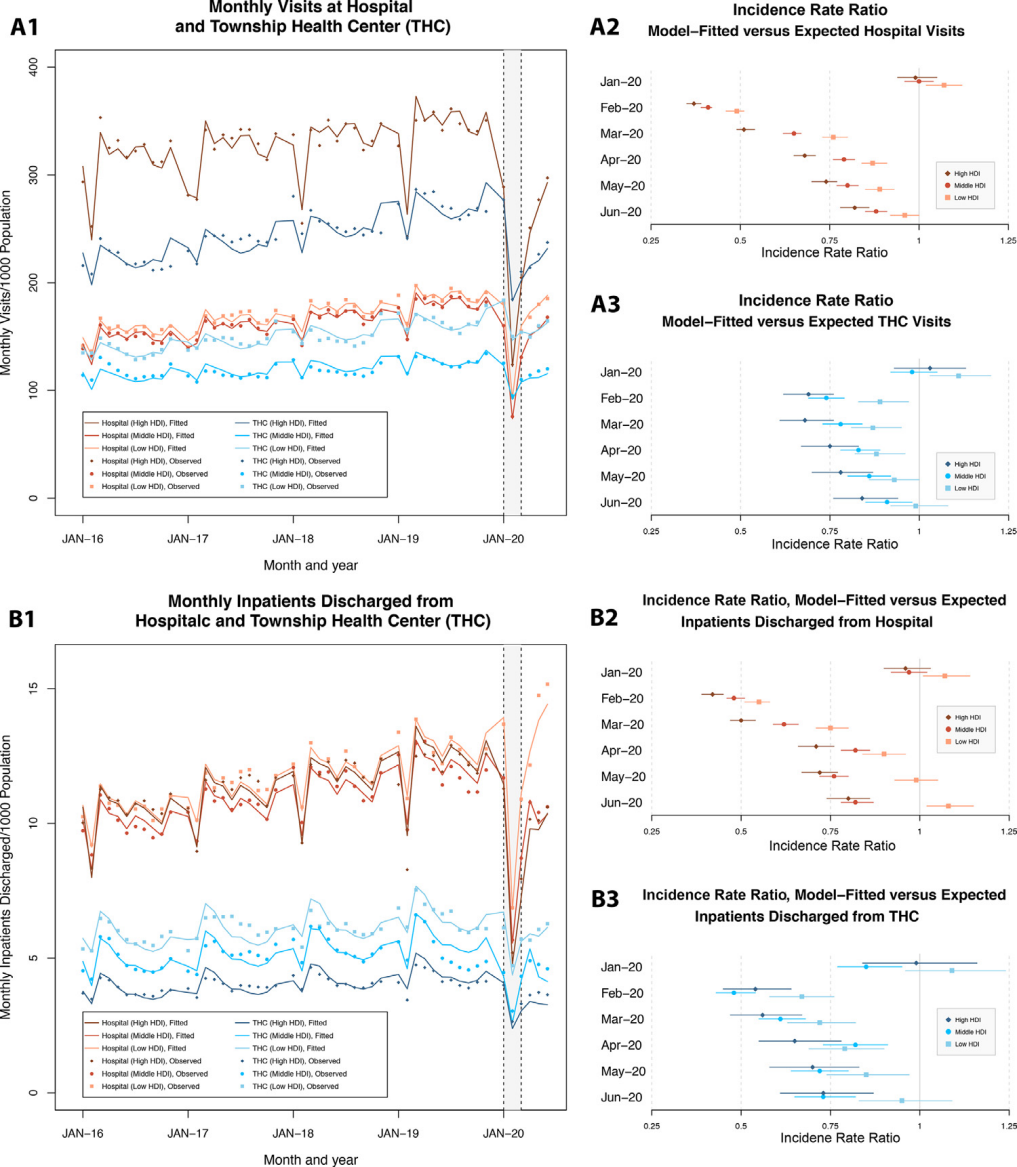
**Monthly health care services utilization rate stratified by HDI group over time.** Panel A1 and B1) Monthly visits (A1) and inpatients discharged (B1) from hospital (red) and township health center (blue). The dots show observed rates and the lines indicate the model-fitted rates. The light, medium and dark shade of the red or blue represents regions with low, middle and high HDI respectively. The gray rectangular shaded area represents the period when over 95% of SARS-COV-2 cases were diagnosed during the study period. Panel A2 and A3) The incidence rate ratio (IRR, model-fitted level versus counterfactual level had the pandemic not occurred) of monthly all-cause visits in hospital (red) and township health center (blue) by HDI category. The diamonds, circles and squares show point estimates of IRR for regions with low, middle and high HDI respectively. The lines indicate 95% confidence interval of the estimates. Panel B2 and B3) The IRR of monthly inpatients discharged from hospital (red) and township health center (blue) by HDI category. The diamonds, circles and squares show point estimates of IRR for regions with low, middle and high HDI respectively. The lines indicate 95% confidence interval of the estimates.

We observed significant decreases in outpatient and inpatient services utilization for health facilities at all levels during the initial pandemic period, in January 2020 (Table 1; Table S1; Fig. 1). The unadjusted reduction in utilization rates in January 2020 was greater (*p*<0.0001) than the reduction in the average utilizations in January months in the pre-COVID period. However, the most precipitous decline occurred in February 2020 for all regions (Fig. 1; Fig. S1), with a 47.6% (range from 24.8% in THC to 70% in primary care clinic) and 47.7% (range from 26.8% in other health facility to 57.8% in first level hospital) decrease in all-cause visits and inpatient volume, respectively. Rebounds were first noted in March 2020 (Fig. 1; Fig. 2; Fig. S1), concurrent with a dramatic drop in new SARS-COV-2 cases and the downgrade of the public health emergency response level from Level I to Level II or III in most provinces as virus threat receded.

### 5.2 Model-based estimates of changes due to the COVID-19 pandemic

The adjusted relative change of the monthly rate of national health service utilization by facility type (hospital versus PHCF) and treatment setting (outpatient and emergency department versus inpatient department) are reported in Table 2. A 51% decrease (IRR: 0.49; 95% CI: 0.48–0.50, *p*<0.0001) in health facility visits was observed in February 2020, greater than the 49% reduction (IRR: 0.51; 95% CI: 0.50–0.54, *p*<0.0001) in inpatient visits, representing a modest (but statistically significant) 6% reduction (ratio of IRR: 0.94; 95% CI: 0.90–0.98, *p* = 0.0079) in the incidence rate ratio. The decreases were greater for hospitals than for PHCFs in both health facility visits (59% versus 44%, *p*<0.0001) and number of inpatients discharged (51% versus 41%, *p*<0.0001). Following the nadir of utilization in February, both health facility visits and inpatient volume showed consistent statistically significant increases from March to June (Fig.1), rising to 89% (95% CI: 86–92%, *p*<0.0001) and 91% (95% CI: 89–93%, *p*<0.0001) of the model-based pre-COVID forecasted (or counterfactual) level had the pandemic not occurred. We estimate a cumulative loss of 1020.5 (95% CI: 951.2- 1089.4; *P*<0.0001) million or 23.9% (95% CI 22.5–25.2%; *P*<0.0001) health facility visits, and 28.9 (95% CI: 26.1–31.6; *P*<0.0001) million or 21.6% (95% CI 19.7–23.4%; *P*<0.0001) inpatients nationwide as of June 2020 due to the SARS-CoV-2 pandemic.

**Table 2:** Comparison of estimated relative change in health care services indicators due to SARS-COV-2 outbreak in hospital and PHCF (January- June 2020)^1^.

	Jan-2020 Initial Pandemic period		Feb-2020 Pandemic period		Mar-2020 Initial Reopening Period		April-2020 Initial Reopening Period		May-2020 Initial Reopening Period		June-2020 Initial Reopening Period	
	IRR^1^ (95% CI)	IRR Ratio^1^ (95% CI)	IRR^1^ (95% CI)	IRR Ratio^1^ (95% CI)	IRR^1^ (95% CI)	IRR Ratio^1^ (95% CI)	IRR^1^ (95% CI)	IRR Ratio^1^ (95% CI)	IRR^1^ (95% CI)	IRR Ratio^1^ (95% CI)	IRR^1^ (95% CI)	IRR Ratio^1^ (95% CI)
***All visits* vs*. Inpatients discharged***												
All visits	0.99 (0.96, 1.02)	1.03 (0.98, 1.09)	0.49 (0.48, 0.50)	0.94 (0.90, 0.98)	0.64 (0.63, 0.65)	0.99 (0.96, 1.01)	0.76 (0.75, 0.77)	0.94 (0.92, 0.97)	0.81 (0.80, 0.82)	0.95 (0.93, 0.98)	0.89 (0.86, 0.92)	0.98 (0.93, 1.02)
Inpatients discharged (Ref)	0.96 (0.92, 1.01)		0.52 (0.50, 0.54)		0.65 (0.64, 0.67)		0.81 (0.79, 0.82)		0.85 (0.83, 0.87)		0.91 (0.89, 0.93)	
***Hospital* vs*. PHCF***												
All visits at Hospital	0.99 (0.95, 1.03)	0.99 (0.95, 1.02)	0.41 (0.39, 0.42)	0.72 (0.70, 0.75)	0.62 (0.61, 0.64)	0.94 (0.92, 0.96)	0.77 (0.75, 0.79)	1.01 (0.99, 1.03)	0.80 (0.79, 0.82)	0.98 (0.97, 0.99)	0.88 (0.87, 0.90)	1.00 (0.98, 1.02)
All visits at PHCF (Ref)	1.00 (0.97, 1.03)		0.56 (0.55, 0.58)		0.66 (0.66, 0.67)		0.76 (0.75, 0.76)		0.82 (0.81, 0.82)		0.88 (0.87, 0.88)	
Inpatients discharged from hospital	0.97 (0.92, 1.02)	1.07 (0.96, 1.20)	0.49 (0.47, 0.51)	0.83 (0.76, 0.90)	0.64 (0.63, 0.65)	0.93 (0.89, 0.97)	0.81 (0.79, 0.83)	1.03 (0.99. 1.05)	0.85 (0.83, 0.87)	0.99 (0.97, 102)	0.92 (0.90, 0.94)	1.02 (0.99, 1.06)
Inpatients discharged from PHCF (Ref)	0.90 (0.82, 0.99)		0.59 (0.54, 0.65)		0.69 (0.66, 0.72)		0.79 (0.77, 0.81)		0.86 (0.84, 0.88)		0.90 (0.87, 0.93)	

1 Derived from negative binomial regression model, adjusting for fixed-effect variable for calendar month, spring festival and catchment population. All visits include outpatient and emergency department visits. PHCF: Primary Health Care Facility.

### 5.3 Heterogeneity in effects across health facilities

The effects were heterogenous across subtypes of hospital and PHCF (Table S2). The reductions in the initial pandemic period was comparable between public and private hospitals for both all-cause visits (59% versus 69% down, *p* = 0.82) and inpatient (51% versus 53% down, *p* = 0.24) volume. In June, inpatient volume increased to 116% (95% CI: 108–125%; *p*<0.0001) of the forecasted level in private hospital but was 14% (95% CI: 12–15%; *p*<0.0001) down in public hospitals. Tertiary hospital showed significantly greater reduction in the initial pandemic period and a weaker rebound in the reopening phase than first level hospitals for both outpatient and inpatient volume. Reduction in visits for primary health care in February ranged from 71% (95% CI: 70–72%; *p*<0.0001) in primary care clinic to 25% (95% CI: 21–28%; *p*<0.0001) in THC, and the reduction in inpatient volume in community health center (56%; 95% CI: 47–63%) was greater (*p*<0.0001) compared with that in THC (39%; 95% CI: 30–38%). Among all indicators, only inpatient volume in private hospital and first level hospital exceeded the forecasted number by June 2020.

### 5.4 Regional heterogeneity in effects

Compared with regions in low the HDI category, more developed regions showed significantly greater reduction in healthcare services utilizations in the initial pandemic period and weaker rebound in the reopening phase (Table 3; Table S3; Fig. 2). In February 2020, a 40% (95% CI: 38–43%, *p*<0.0001) model fitted decrease was observed in hospital and THC visits for regions with low HDI, significantly lower (*p*<0.0001 and *p*<0.0001) than the 52% (95% CI: 50–54%, *p*<0.0001) and 55% (95% CI: 53–58%, *p*<0.0001) decreases for regions in middle and high HDI groups, respectively. Similarly, the 43% (95% CI: 39–46%, *p*<0.0001) decrease in monthly inpatients discharged from hospital and THC in regions of low HDI was lower (*p*<0.0001 and *p*<0.0001) than the 51% (95% CI: 48–53%, *p*<0.0001) and 53% (95% CI: 49–56%, *p*<0.0001) decrease for regions in middle and high HDI group respectively. By June 2020, utilization levels for all indicators were comparable to or higher than expected levels in regions with low HDI, but still significantly lower than expected levels in more developed regions, ranging from 27% (95% CI: 13–39%, *p* = 0.0007) down for inpatient volume at THC in regions with high HDI to 9% (95% CI: 2–15%, *p* = 0.0084) down for THC visits in regions with middle HDI (Table S3).

**Table 3:** Estimated relative change in health care services utilization indicators in hospital and THC due to SARS-COV-2 outbreak, stratified by HDI groups+ and treatment settings.

	Jan-2020 Initial Pandemic period		Feb-2020 Pandemic period		Mar-2020 Initial Reopening Period		April-2020 Initial Reopening Period		May-2020 Initial Reopening Period		June-2020 Initial Reopening Period	
	IRR^1^ (95% CI)	IRR Ratio^1^ (95% CI)	IRR^1^ (95% CI)	IRR Ratio^1^ (95% CI)	IRR^1^ (95% CI)	IRR Ratio^1^ (95% CI)	IRR^1^ (95% CI)	IRR Ratio^1^ (95% CI)	IRR^1^ (95% CI)	IRR Ratio^1^ (95% CI)	IRR^1^ (95% CI)	IRR Ratio^1^ (95% CI)
Number of visits^2^												
Overall	.99 (.96, 1.04)		.49 (.47, .50)		.66 (.65, .67)		.78 (.76, .80)		.83 (.82, .84)		.89 (.88, .91)	
Low HDI (REF)	1.08 (1.04, 1.14)		.60 (.57, .62)		.79 (.76, .83)		.88 (.84, .92)		.90 (.86, .94)		.97 (.93, 1.01)	
Middle HDI	1.00 (.96, 1.04)	.92 (.87, .97)	.48 (.46, .50)	.81 (.76, .85)	.68 (.65, .70)	.85 (.81, .90)	.80 (.77, .83)	.91 (.86, .96)	.81 (.78, .85)	.90 (.86, .96)	.89 (.85, .92)	.92 (0.87, .97)
High HDI	1.00 (.94, 1.06)	.92 (0,96, .99)	.45 (.42, .47)	.75 (.70, .81)	.58 (.55, .62)	.74 (.69, .79)	.72 (68, .77)	.82 (.77, .89)	.77 (.73, .82)	.86 (.80, .92)	.85 (.80, .90)	.87 (.81, .94)
Inpatients discharged^3^												
Overall	.96 (.92, 1.01)		.51 (.49, .53)		.65 (.64, .66)		.81 (.79, .83)		.85 (.83, .87)		.92 (.90, .94)	
Low HDI (REF)	1.07 (1.01, 1.14)		.57 (.54, .61)		.75 (.71, .80)		.88 (.83, .94)		.98 (.92, 1.04)		1.07 (1.01, 1.14)	
Middle HDI	.97 (.92, 1.02)	.90 (.84, .97)	.49 (.47, .52)	.86 (.80, .93)	.63 (.59, .66)	.83 (.77, .90)	.81 (.77, .85)	.92 (.85, .99)	.76 (.72, .80)	.77 (.72, .84)	.82 (.78, .86)	.76 (.71, .83)
High HDI	.99 (.91, 1.07)	.92 (.84, 1.01)	.47 (.44, .51)	.83 (.75, .91)	.57 (.53, .62)	.76 (.69, .84)	.77 (.71, .83)	.87 (.79, .96)	.76 (.70, .83)	.78 (.71, .86)	.84 (.78, .91)	.79 (.71, .87)

1 Derived from negative binomial regression model, adjusting for fixed-effect variable for calendar month, spring festival and catchment population, random intercepts and slopes by province.

2 Number of visits in hospitals and township health centers.

3 Inpatients discharged from hospitals and township health centers.

+ HDI _high_: Beijing, Shanghai, Tianjin, Jiangsu, Zhejiang, Liaoning, Zhejiang; HDI _middle_: Shandong, Jilin, Inner Mongolia, Fujian, Shaanxi, Hunan, Hebei, Henan, Jiangxi; HDI _low_: Ningxia, Anhui, Sichuan, Guangxi, Xinjiang, Qinghai, Gansu, Guizhou, Yunnan.

## 6 Discussion

The results of our analysis indicate substantial reductions in the monthly volume of health facility visits and inpatient discharges in early 2020, as well as a progressive restoration following the nadir of health service utilization in February 2020, coinciding with the initial SARS-COV-2 outbreak and the subsequent control of the outbreak, respectively. These patterns were apparent for all types of health facilities and throughout the country, even in provinces where very few cases occurred. Utilization rates had not recovered to their pre-SARS-COV-2 levels by June for most indicators, despite no new cases reported in most provinces.

The abrupt reduction in the number of health facility visits was likely attributable to demand, supply and physical accessibility factors associated with the SARS-COV-2 outbreak [13]. First, the lack of knowledge of the virus and the fear of contracting the disease made patients and their family members less likely to visit health facilities [9,^13^,^15^,18]. Patients may have chosen to forgo or delay in-person care at health facilities during the pandemic even when the lockdown policies were lifted [13,18]. Although there were no additional confirmed cases in most provinces after March 2020, awareness of the ongoing sporadic transmissions mainly caused by imported and sporadic cases elsewhere in the country might have continued to influence health seeking behaviors. Also, there was likely a conversion of many in-person visits to telehealth visits and consultations [39], [40], [41], [42], [43]. Due to the quarantine and social distancing need, there has been a surge in the use of telehealth and virtual care services provided by traditional health facilities or through the platforms of technology companies in China during the pandemic, which has been widely reported [43], [44], [45], [46]. Second, the drop of income and employer-based health insurance coverage due to furlough or loss of job further raised the barriers to accessing healthcare services [47], [48], [49]. The financial and social security of the less-educated, low-income and low-skilled groups, rural-hukou migrant workers in particular, have been disproportionately affected because of the substantially higher unemployment rate among them at this crisis, exacerbating the pre-existing inequalities in access to health services [48,50]. Third, restrictions on mobility were implemented extensively across the country. Following Wuhan's lockdown on 23 January, all provinces subsequently initiated the Level I public health emergency response by the end of January. By 4 February, over 95% of cities in each province had implemented transmission control measures either before or after the first case occurred [7,8]. About 40% of the cities suspended intra-city public transportation (bus and subway) and 64% of the cities prohibited travel to and from any other cities by any means [7]. Fourth, providers may delay elective health care to reduce the risk of transmitting the virus to either patients or health care workers despite most health facilities remaining open [13,18]. In some provinces, health facilities or their outpatient services for non-communicable diseases and non-emergency surgeries were suspended [8,^18^,51]. Finally, China's rapid and strict countermeasures not only effectively controlled the spread of SARS-COV-2, but also significantly reduced the incidence and mortality of non-SARS-COV-2 diseases including cardiovascular disease, non-SARS-COV-2 pneumonia, chronic respiratory diseases and injuries, and the healthcare utilization typically associated with these conditions [6].

The larger reduction in more developed regions and in higher level health facilities also supports the hypothesis that the widespread reduction in health care utilization in China was attributable to the SARS-COV-2 epidemic. Due to the low number of confirmed cases and the successful mitigation of the transmission, more provinces in the lower HDI group downgraded their public health emergency response level from level I to Level II or III in February [3,^7^,^52^,53], resuming intercity travel and intracity public transport. Provinces with lower HDI are more likely to have greater rural populations. Given media coverage about SARS-COV-2 prevention that focused predominantly on large urban cities, rural residents reported less positive attitudes toward the effectiveness of performing preventive behaviors; as such, rural residents were less likely to engage in behaviors such as avoiding gathering, staying home as much as possible and avoiding public transportation [10]. Moreover, the inter-city and inter-provincial travel ban was more likely to prevent patients from rural and less developed regions from seeking health care at health facilities in more developed regions. The urge to implement hierarchical medical care to avoid crowds in tertiary hospitals during the epidemic may also partially explain the greater losses of visits in tertiary hospitals.

We found that nearly all healthcare utilization measures had not recovered to the pre-SARS-COV-2 levels by June 2020, despite very few new SARS-COV-2 cases since April and the removal of travel restrictions across the country in March 2020 [3,54]. These reductions and stagnations in prevention and treatment will likely have significant collateral effects on population health that greatly exceed the direct health effects from the infection [9,^16^,^17^,^26^,55]. In China, only a small portion of the unemployed receive unemployment insurance. Despite some flexibility (indicated by the government in May) in offering one-time subsidies to the unemployed not enrolled in the unemployment insurance system, the new policy applied only to a very limited number of exceptional cases [48]. Crippling losses in revenue owing to the precipitous decline of patient visits threaten the viability of a substantial number of healthcare facilities and providers, especially private primary care clinics and village practices that were already financially vulnerable [49]. In this context, our findings call for renewed efforts to educate patients about safety protocols, as well as targeted interventions to restore community trust and confidence in health facilities [13,14]. Also, our findings suggest that flexible and broad-based compensation programs may be needed to alleviate financial barriers to accessing healthcare services among vulnerable populations [48,49]. The findings also suggest the need to adopt innovative payment models such as a capitation model that decouples compensation from the volume of services provided; such approaches could help stabilize facility and provider finances compared to the current fee-for-service system, especially in the extreme circumstance of a pandemic [49].

To our knowledge, this study is the first to quantify the effect of the SARS-COV-2 outbreak on longitudinal trends in health services utilization in China, where the disease was first reported. Our analyses are comprehensive, with detailed monthly data spanning multiple years before, during, and after the outbreak, covering the entire population, and using robust statistical methods. These analyses show the value of routine health information system (RHIS) data for timely and effectively tracking the performance of health system in low- and middle-income countries [17,56].

Our results are subject to several important limitations. First, the possibility of data collection errors and delay in reporting cannot be ruled out even though routine data were extracted and reported using standardized procedures. If so, the use of the aggregate RHIS data may result in some bias in the estimates. Second, given the nature of the aggregated data, we were not able to explore the cross-province heterogeneity by health facility type, nor were examinations by patient-level demographic variables (sex and age) or disease type feasible. Third, we were not able to estimate the utilization losses in Hubei in the first two months of the outbreak, January and February 2020, due to non-reporting of the outcomes. However, the exclusion of Hubei in the analysis at national and subnational level would most likely not significantly affect the results because that patient volume in Hubei in a typical month accounted for less than 4% of the national total and the recovery patterns for both outpatient and inpatient volume in Hubei after February were comparable to those in other regions that had implemented similar public health emergency response (Fig. S1). Also, tests for interaction have notably lower power than tests for main effects [57]; thus analyses of differential trends between groups that lack statistical significance may represent false negative findings. Last, our assessment of health services utilization could not capture trends in the use of telehealth visits and consultations, nor were we able to quantify potential changes in the quality of care before, during and after the SARS-COV-2 outbreak.

In conclusion, our study shows that outpatient and inpatient volume in healthcare facilities at all levels significantly declined in conjunction with the SARS-COV-2 outbreak, and most utilization measures have not returned to their pre-outbreak levels as of June 2020, despite the fact that China rapidly and effectively controlled the pandemic. Sustained monitoring and targeted interventions that restore public trust and confidence in healthcare facilities and protect providers’ financial risk are needed to mitigate the collateral disruption on healthcare services. Future research is needed to establish which vulnerable patient groups and which disease-specific healthcare services were most affected by the pandemic, in order to continue to inform strategies and policies aimed at mitigating the impact of potential future pandemics.

## Contributors

HX, YG and JMU conceived the research question and developed the protocol. YG and JMU oversaw study implementation. HX was responsible for the data curation and analysis and the writing of the first draft of the manuscript. XD, BHW, FL, OA, YG and JMU assisted with development of the study design, data analysis, data interpretation, and critical review of the manuscript. HX, YG and FL have assessed and verified the underlying data and take responsibility for the integrity of the data and the accuracy of the data analysis. All authors read and approved the final manuscript.

## Data sharing statement

The data that supports the findings of this research are publicly available from the Center for Health Statistics and Information: http://www.nhc.gov.cn/mohwsbwstjxxzx/s2906/new_list.shtml.

## Funding

The National Natural Science Foundation of China (No. 72042014).

## Declaration of Competing Interest

The authors declare no competing interests related to the study.

## Appendix Supplementary materials

Image, application 1

Image, application 2
