# Supplementary figures and images for: The impact of the COVID-19 pandemic on health services utilization in China: Time-series analyses for 2016–2020
Source: Lancet Reg Health West Pac. 2021 Mar 24;9:100122. doi: 10.1016/j.lanwpc.2021.100122 (PMC8315657; doi:10.1016/j.lanwpc.2021.100122)

# Hospital Visits

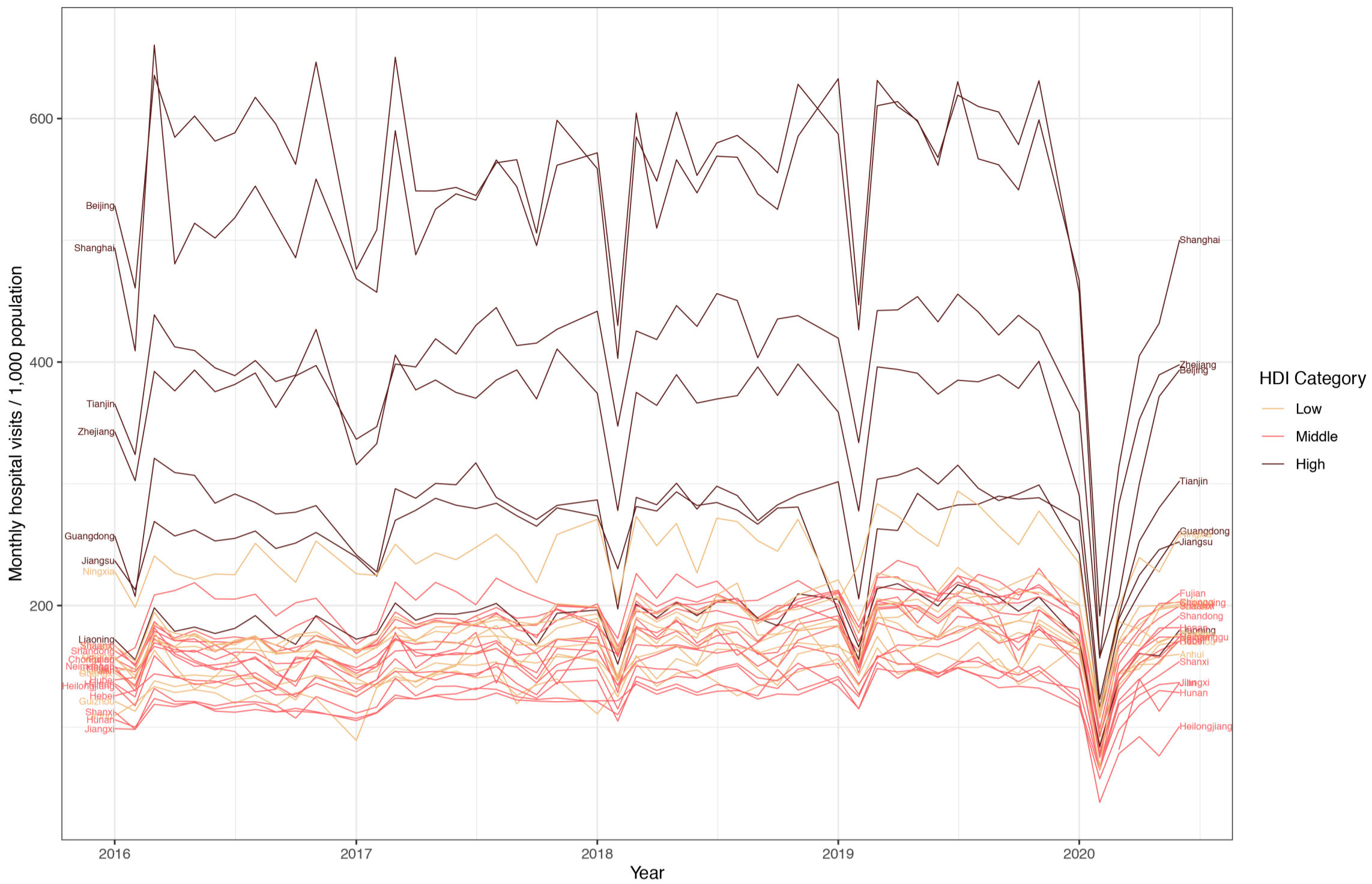

# Inpatient Discharged

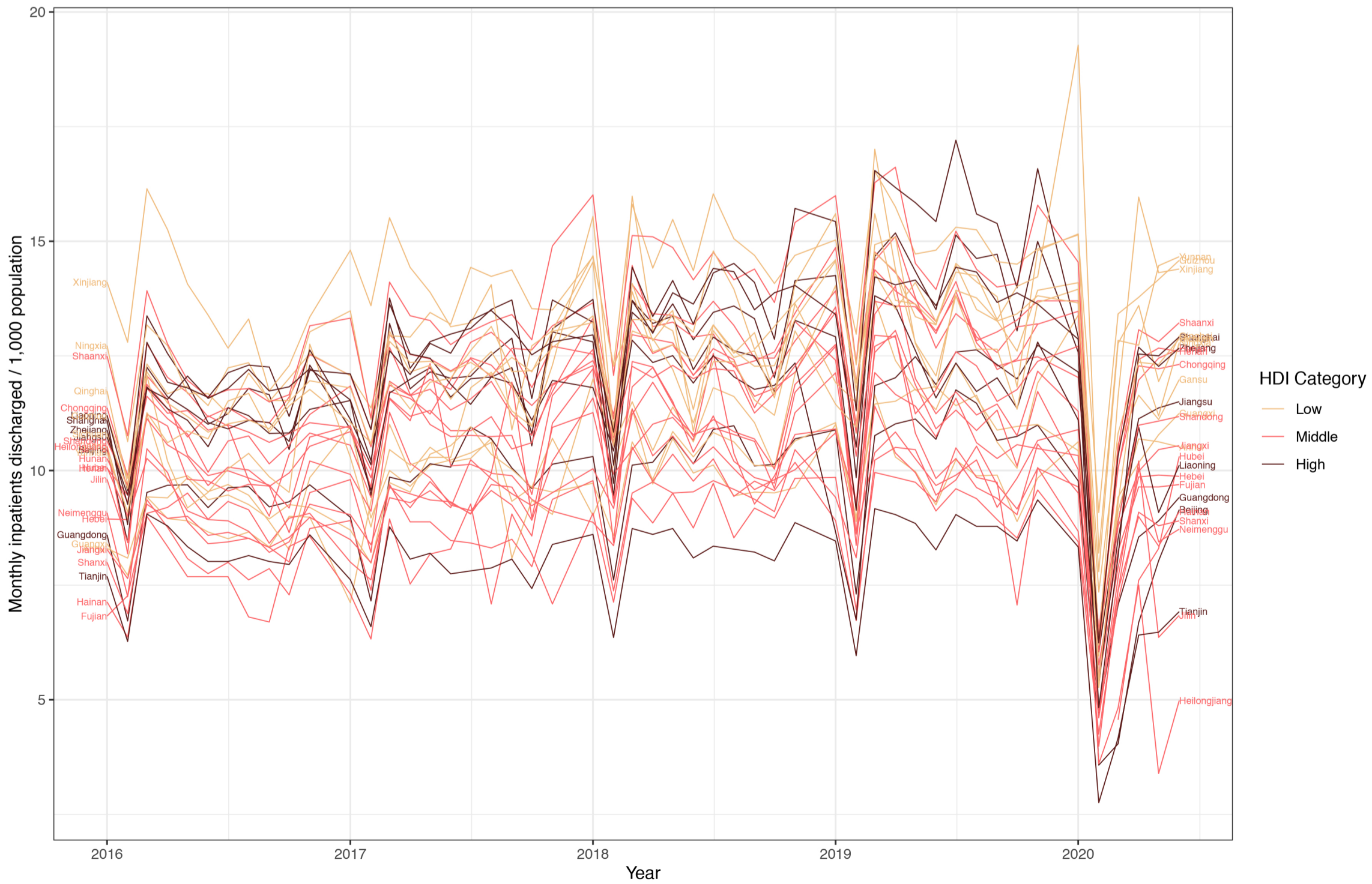

Supplement: Supplementary file 1 [file mmc1.pdf]
