# Supplementary material for: The impact of the COVID-19 pandemic on health services utilization in China: Time-series analyses for 2016–2020
Source: Lancet Reg Health West Pac. 2021 Mar 24;9:100122. doi: 10.1016/j.lanwpc.2021.100122 (PMC8315657; doi:10.1016/j.lanwpc.2021.100122)
Supplement: Supplementary file 2 [file mmc2.docx]

STROBE Statement—checklist of items that should be included in reports of observational studies

|  | Item No. | Recommendation | Page  No. | Relevant text from manuscript |
| --- | --- | --- | --- | --- |
| **Title and abstract** | 1 | (*a*) Indicate the study’s design with a commonly used term in the title or the abstract | 1 | “We conducted a retrospective observational cohort study of health services utilization from health facilities at all levels in all provinces of mainland China” |
|  |  | (*b*) Provide in the abstract an informative and balanced summary of what was done and what was found | 1 | “We analyzed monthly all-cause health facility …provincial Human Development Index (HDI)” |
| Introduction | | | |  |
| Background/rationale | 2 | Explain the scientific background and rationale for the investigation being reported | 3 | “Responding to an outbreak of SARS-COV-2 in December 2019, China implemented a range of behavioural… stringent restrictions on mobility” |
| Objectives | 3 | State specific objectives, including any prespecified hypotheses | 3, 4 | “The aim of the present study is to…treatment setting ”, “The specific hypotheses we intended to test include… treatment setting?” |
| Methods | | | |  |
| Study design | 4 | Present key elements of study design early in the paper | 4 | “We conducted an interrupted time series analysis to examine the impact of SARS-COV-2 on healthcare services utilization” |
| Setting | 5 | Describe the setting, locations, and relevant dates, including periods of recruitment, exposure, follow-up, and data collection | 3 | “We extracted all available data for monthly facility-based health services utilization from the routine health information system of the Center for Health Statistics and Information, National Health Commission of China for the period from January, 2016, to June, 2020.” |
| Participants | 6 | (*a*) *Cohort study*—Give the eligibility criteria, and the sources and methods of selection of participants. Describe methods of follow-up  *Case-control study*—Give the eligibility criteria, and the sources and methods of case ascertainment and control selection. Give the rationale for the choice of cases and controls  *Cross-sectional study*—Give the eligibility criteria, and the sources and methods of selection of participants |  |  |
|  |  | (*b*) *Cohort study*—For matched studies, give matching criteria and number of exposed and unexposed  *Case-control study*—For matched studies, give matching criteria and the number of controls per case |  |  |
| Variables | 7 | Clearly define all outcomes, exposures, predictors, potential confounders, and effect modifiers. Give diagnostic criteria, if applicable | 5 | “Here, *Y_it_* denotes the value of one of the response variables (e.g., monthly outpatient visits at hospitals) included in this study for province *i* at time *t*… *P_it_* is the catchment population for hospitals or THCs in province *i* at time *t*. ” |
| Data sources/ measurement | 8* | For each variable of interest, give sources of data and details of methods of assessment (measurement). Describe comparability of assessment methods if there is more than one group | *4* | *“*Subnational level human development index (HDI) was retrieved from China National Human Development Report 2019 to reflect regional-level SES…Yearly urban and rural population estimates by region were extracted from China Statistical Yearbooks.” |
| Bias | 9 | Describe any efforts to address potential sources of bias | 4, 5 | “Provincial Health Commissions verify and review the data at the primary level... The National Health Commission oversees and checks the quality of data …in the reporting process”, “*SF_t_* is the number of days of spring festival holiday in month t. *Month* is the indicator of calendar month of the year with month of January as the reference category. *P_it_* is the catchment population for hospitals or THCs in province *i* at time *t*. ” |
| Study size | 10 | Explain how the study size was arrived at |  |  |

Continued on next page

| Quantitative variables | 11 | Explain how quantitative variables were handled in the analyses. If applicable, describe which groupings were chosen and why |  |  |
| --- | --- | --- | --- | --- |
| Statistical methods | 12 | (*a*) Describe all statistical methods, including those used to control for confounding | 4, 5 |  |
|  |  | (*b*) Describe any methods used to examine subgroups and interactions | 5 |  |
|  |  | (*c*) Explain how missing data were addressed | 5 | “Tibet and Hubei and Tibet were excluded from the subnational analysis due to missing data in the pre- and post-outbreak (January and February 2020) period respectively. No missing data points or extreme outliers were found in other provinces after visually inspecting the data by indicator, health facility type and region.” |
|  |  | (*d*) *Cohort study*—If applicable, explain how loss to follow-up was addressed  *Case-control study*—If applicable, explain how matching of cases and controls was addressed  *Cross-sectional study*—If applicable, describe analytical methods taking account of sampling strategy |  |  |
|  |  | (*e*) Describe any sensitivity analyses |  |  |
| Results | | | | |
| Participants | 13* | (a) Report numbers of individuals at each stage of study—eg numbers potentially eligible, examined for eligibility, confirmed eligible, included in the study, completing follow-up, and analysed |  |  |
|  |  | (b) Give reasons for non-participation at each stage |  |  |
|  |  | (c) Consider use of a flow diagram |  |  |
| Descriptive data | 14* | (a) Give characteristics of study participants (eg demographic, clinical, social) and information on exposures and potential confounders |  |  |
|  |  | (b) Indicate number of participants with missing data for each variable of interest |  |  |
|  |  | (c) *Cohort study*—Summarise follow-up time (eg, average and total amount) | 4 | “We extracted all available data for monthly facility-based health services utilization from the routine health information system of the Center for Health Statistics and Information, National Health Commission of China for the period from January, 2016, to June, 2020” |
| Outcome data | 15* | *Cohort study*—Report numbers of outcome events or summary measures over time | *6* | *“*The monthly all cause visits and inpatients discharged increased by 18% ((110, 505, 000)/618, 623, 000) and 26% (4, 436, 000/21, 454, 000) respectively from January 2016 to December 2019.*”* |
|  |  | *Case-control study—*Report numbers in each exposure category, or summary measures of exposure |  |  |
|  |  | *Cross-sectional study—*Report numbers of outcome events or summary measures |  |  |
| Main results | 16 | (*a*) Give unadjusted estimates and, if applicable, confounder-adjusted estimates and their precision (eg, 95% confidence interval). Make clear which confounders were adjusted for and why they were included | 6,7 | “Model-based estimates of changes due to the SARS-COV-2 pandemic” |
|  |  | (*b*) Report category boundaries when continuous variables were categorized |  |  |
|  |  | (*c*) If relevant, consider translating estimates of relative risk into absolute risk for a meaningful time period |  |  |

Continued on next page

| Other analyses | 17 | Report other analyses done—eg analyses of subgroups and interactions, and sensitivity analyses | 6, 7 | “Heterogeneity in effects across health facilities”, “Regional heterogeneity in effects” |
| --- | --- | --- | --- | --- |
| Discussion | | | | |
| Key results | 18 | Summarise key results with reference to study objectives | 8 | “The results of our analysis indicate substantial reductions in the monthly volume of health facility visits and inpatient discharges …Utilization rates had not recovered to their pre-SARS-COV-2 levels by June for most indicators, despite no new cases reported in most provinces.” |
| Limitations | 19 | Discuss limitations of the study, taking into account sources of potential bias or imprecision. Discuss both direction and magnitude of any potential bias | 10 | “Our results are subject to several important limitations. …Last, our assessment of health service utilization could not capture trends in the use of telehealth visits and consultations, nor were we able to quantify potential changes in the quality of care before, during and after the SARS-COV-2 outbreak.  ” |
| Interpretation | 20 | Give a cautious overall interpretation of results considering objectives, limitations, multiplicity of analyses, results from similar studies, and other relevant evidence | 1, 10 | “In conclusion, our study shows that outpatient and inpatient volume in healthcare facilities at all levels significantly declined in conjunction with the SARS-COV-2 outbreak…in order to continue to inform strategies and policies aimed at mitigating the impact of potential future pandemics.  ” |
| Generalisability | 21 | Discuss the generalisability (external validity) of the study results |  |  |
| Other information | |  | | |
| Funding | 22 | Give the source of funding and the role of the funders for the present study and, if applicable, for the original study on which the present article is based | 6, 11 | “The funder of the study had no role in study design, data collection, data analysis, data interpretation, or writing of the report. The corresponding author had full access to all the data in the study and had final responsibility for the decision to submit for publication.  ” |

*Give information separately for cases and controls in case-control studies and, if applicable, for exposed and unexposed groups in cohort and cross-sectional studies.

**Note:** An Explanation and Elaboration article discusses each checklist item and gives methodological background and published examples of transparent reporting. The STROBE checklist is best used in conjunction with this article (freely available on the Web sites of PLoS Medicine at http://www.plosmedicine.org/, Annals of Internal Medicine at http://www.annals.org/, and Epidemiology at http://www.epidem.com/). Information on the STROBE Initiative is available at www.strobe-statement.org.
